# Endocrine-disrupting effects of environmental BPS and PFOS on human brain organoid development

**DOI:** 10.3389/fendo.2025.1692333

**Published:** 2025-12-04

**Authors:** Andrea Di Credico, Giulia Gaggi, Sandra Bibbò, Timothy A. Blenkinsop, Angela Di Baldassarre, Barbara Ghinassi

**Affiliations:** 1 Department of Medicine and Aging Sciences, G. d'Annunzio University of Chieti-Pescara, Chieti, Italy; 2 Cell Reprogramming and Differentiation Lab, Center of Advanced Studies and Technologies (CAST), G. d'Annunzio University of Chieti-Pescara, Chieti, Italy; 3 UdA-Tech Lab, G. D’Annunzio University of Chieti-Pescara, Chieti, Italy; 4 Department of Ophthalmology, Cell Development and Regenerative Biology, Black Family Stem Cell Institute, Icahn School of Medicine at Mount Sinai, New York, NY, United States; 5 Department of Innovative Technologies in Medicine and Dentistry, G. D’Annunzio University of Chieti-Pescara, Chieti, Italy

**Keywords:** brain organoids, neurodevelopment, neural differentiation, endocrine disrupting chemicals, BPS, PFOS

## Abstract

**Objective:**

Prenatal exposure to environmental endocrine-disrupting chemicals (EDCs) has been increasingly linked to neurodevelopmental impairment. Bisphenol S (BPS) and perfluoro-octane sulfonate (PFOS), two widely distributed EDCs detected in maternal and fetal tissues, raise concern due to their potential to interfere with brain development even at low environmental doses.

**Methods:**

a phenotypic screening on human iPSC-derived cerebral organoids was performed to explore whether chronic exposure to BPS and PFOS could affect key neurodevelopmental processes.

**Results:**

Both compounds affected key neurodevelopmental processes, including neuronal proliferation, cortical specification, synaptogenesis, glutamatergic differentiation, mitochondrial function, and choroid plexus formation. Importantly, TUNEL assay confirmed the absence of significant cytotoxicity. BPS exposure was associated with reduced ERβ, GPER, and phosphorylated Akt expression, suggesting a possible involvement of estrogen-related pathways. PFOS exposure coincided with decreased transthyretin expression, suggesting a potential influence on thyroid hormone availability.

**Conclusions:**

Exposure to multiple EDCs may disrupt distinct endocrine axes, producing cumulative impacts on human brain development. These findings underscore the value of human-relevant models for identifying endocrine-mediated neurodevelopmental hazards. While the observed molecular changes suggest distinct hormonal pathways may be involved, future mechanistic studies, including co-exposures with receptor modulators, will be required to establish causal relationships.

## Introduction

Human brain development is a tightly regulated process that begins in early embryogenesis and extends into young adulthood (1). It involves sequential phases of neurogenesis, neuronal migration, and synaptogenesis, which are essential for the formation of functional neural circuits underlying cognition, sensory processing, and behavior (1–3).

Disruptions during these critical stages can have long-lasting consequences, contributing to neurodevelopmental disorders such as autism spectrum disorder (ASD), attention-deficit/hyperactivity disorder (ADHD), and intellectual disabilities (4).

Among the environmental factors implicated in altered neurodevelopment, endocrine-disrupting chemicals (EDCs) have emerged as compounds of concern. Bisphenol S (BPS) and perfluoro-octane sulfonate (PFOS) are two widespread EDCs found in consumer products including plastics, thermal receipts, non-stick coatings, and personal care items (5–8).Human biomonitoring studies have documented measurable PFOS levels in Italian populations (9, 10) and detectable urinary BPS in a European adult cohort (11). Although these data derive from separate studies, together they support a plausible real-life scenario in which individuals are chronically exposed to both compounds. Investigating BPS and PFOS in combination at environmentally relevant, low-dose levels reflects this realistic exposure pattern, while also considering their mechanistic complementarity, BPS modulating estrogen-receptor pathways (12–14) and PFOS disrupting thyroid-hormone signaling (15) which may yield additive or synergistic neurodevelopmental effects.

From a regulatory perspective, several frameworks, including the EU REACH Regulation and international guidance documents, have incorporated assays addressing EDC mechanisms, particularly those linked to estrogen receptor (ER) or thyroid hormone receptor signaling (16). However, most validated *in vitro* and *in vivo* tests still focus on receptor binding, hormone synthesis, or systemic hormone levels, without directly assessing neurodevelopmental endpoints (17). Consequently, human-relevant models that capture hormone-mediated effects on brain development remain scarce, despite the increasing recognition of these pathways as critical determinants of developmental neurotoxicity (18).

Estrogen and thyroid hormone signaling play pivotal roles in early brain development. Estrogen receptors (ERα, ERβ, and the G-protein–coupled estrogen receptor, GPER) influence neural stem cell proliferation, differentiation, and synaptogenesis (19). Similarly, thyroid hormones act through nuclear receptors to regulate neural progenitor proliferation, neuronal migration, synaptic maturation, and oligodendrocyte differentiation (20, 21).

Disruption of these hormone-regulated pathways during sensitive developmental windows has been associated with cognitive impairment, neurobehavioral abnormalities, and defective myelination (20, 22).

Notably, both chemicals can cross the human placenta during pregnancy (23), a period characterized by high sensitivity to hormonal cues. Yet, despite growing concern (24–26), the specific mechanisms by which these EDCs may impact the human brain remain poorly understood. Most studies to date rely on animal models or high-dose, short-term exposures that do not reflect real-life scenarios (27, 28), and often fail to capture the complexity of human neurodevelopment (29).

The ability of stem cells to generate diverse cell types offers great potential to model several diseases (30–35), and the development of brain organoids derived from human induced pluripotent stem cells (hiPSCs) offers a powerful platform to model early brain formation in a human-relevant context (36, 37). These three-dimensional structures mimic the architecture and functionality of the human brain on a miniature scale (38), offering an accurate representation of the complex cellular interactions and developmental processes that occur *in vivo* (39, 40).In this study, we employed brain organoids to investigate the effects of chronic exposure to environmental doses of BPS and PFOS, individually and in combination, on human neurodevelopment. We examined a range of morphological and molecular parameters, including progenitor proliferation, cortical specification, synaptogenesis, glutamatergic differentiation, mitochondrial function, and hormone receptor–related signaling. Our findings aim to provide mechanistic insights into how real-life exposures to persistent pollutants may compromise brain development and inform ongoing efforts in environmental health risk assessment.

## Materials and methods

### Human iPSCs maintenance and culture

Two lines of hiPSCs from male donors (41), and (30HU-002, iXcell Biotechnology) were cultured on a monolayer of irradiated embryonic murine fibroblasts (A24903, Thermo Fischer Scientific, Waltham, MA, USA) in Dulbecco’s Modified Eagle’s Medium/Nutrient Mixture F-12 (DMEM/F12, 11320033, Gibco) supplemented with 20% Knockout Serum Replacement (KO), 1% penicillin/streptomycin, 2 mM l-glutamine, 1% MEM nonessential amino acid, 55μM β-Mercaptoethanol (all Life Technologies) and 5 ng/mL basic fibroblast growth factor (bFGF) (R&D Systems) or in Matrigel (CLS354263, Corning, Sommerville, MA, USA)-coated plates using mTesR plus medium (Stemcell Technologies). hiPSCs were maintained at 37 °C, 5% CO_2_. iPSCs were split every 5 days using 1x EDTA, and 10 µM ROCK Inhibitor Y-27632.

The hiPSC lines underwent standard banking and quality control prior to use. Banking was performed at early passages under controlled cryopreservation conditions using KO serum in 10%DMSO. Quality control included verification of pluripotency marker expression by immunofluorescence and mycoplasma testing. Only hiPSC batches passing all quality control criteria were expanded and used for subsequent differentiation experiments.

### Cerebral organoids differentiation protocol and EDCs treatment

Cerebral organoids were obtained using the STEMdiff Cerebral Organoid kit (STEMCELL TECHNOLOGIES, Vancouver, Canada) based on the protocol published by Lancaster et al. (36). Briefly, on Day 0, hiPSCs were seeded in 96-well round-bottom ultra-low attachment microplate (Corning, Sommerville, MA, USA) in Seeding Medium (Basal Medium 1 + Supplement A + 10 µM ROCK Inhibitor Y-27632). Cells were cultured in Organoid Formation Medium (Basal Medium 1 + Supplement A), and then from Day 5 in a Neuronal induction medium (Basal Medium 1 + Supplement B) in 24-well ultra-low attachment plate (Corning 3473). On day 7, organoids were embedded in Matrigel drop (CLS354263, Corning, Sommerville, MA, USA) and incubated in Expansion Medium (Basal Medium 2 + Supplement C and D) in 6-Well Ultra-Low Adherent (100-0083). From day 10 onwards, organoids were cultured in Maturation Medium (Basal Medium 2 + supplement E) and shaken continuously at 65 rpm using Celltron (INFORS HT, I69222). To study the EDCs effects, organoids were chronically treated with 50nM BPS, and/or 100nM of PFOS (Wellington Laboratories Inc., Canada) and vehicle (CTRL, methanol 0.129%, a concentration that has been shown to be non-toxic in previous studies (24, 42)) from day 10 onward. Medium change with EDCs was performed every 3 days. On days 20 and 40, cerebral organoids were collected and analyzed. Any organoids that showed leakage from Matrigel or that had fused together were excluded for subsequent analysis.

### Organoids imaging: morphological, morphometrical and immunofluorescent analyses

For morphometrical analysis, brain organoid brightfield images were acquired using EVOS M7000 during the maturation phase at different timepoints. The area was measured by Fiji (ImageJ) software version 1.54k using the “Threshold” function, while the shape analysis was performed by measuring the organoid circularity (circularity index: 4 π*area/perimeter).

For the other analyses, brain organoids were embedded in optimal cutting temperature (OCT) compound (Leica, Wetzlar, Germany) then frozen at -80 °C, and 10 μm thick sections were obtained using the CM1950 cryostat (Leica, Wetzlar, Germany).

For hematoxylin and eosin staining, slides were fixed in acetone for 5 min, incubated in Mayer’s hemalum solution (TC62200QQ, Titolchimica) for 1 min and, after washings, with eosin for 45 sec. Slides were mounted with Histo mount medium and the ventricular-like zone (VLZ) area was measured using Fiji (ImageJ) software version 1.54k: the outer edges of VLZ were identified; then the internal empty area was subtracted to obtain the actual VLZ area expressed in µm^2^.

For immunofluorescent staining, cryosections were fixed in acetone, permeabilized with 0.5% Triton for 15 min and blocked in 5% BSA for 30 min. The following primary antibodies were used for immunofluorescence staining: anti-Ki-67 (53-5698-82, Thermo Fisher Scientific, MA, US), anti-synaptophysin (SAB4502906, Sigma-Aldrich, St. Louis, Missouri, US), anti-TBR1 (WH0010716M1, Sigma-Aldrich, St. Louis, Missouri, US), anti-Ctip2 (ab233713, Abcam, Cambridge, UK), anti-SATB2 (ab34735, Abcam, Cambridge, UK), anti-transthyretin (MA5-32634, Thermo Fisher Scientific, MA, US), anti-β-Tubulin III (T8578, Sigma-Aldrich, St. Louis, Missouri, US), anti-NMDAR2B (ab65783, Abcam, Cambridge, UK), anti-VGlutT1(ab180188, Abcam, Cambridge, UK), and anti-PSD-95 (ab18258, Abcam, Cambridge, UK). Immunofluorescent images were acquired using Operetta CLS (Revvity). Due to limitations in nuclear segmentation accuracy within densely proliferative zones, Ki-67 expression was quantified as the ratio of Ki-67+ area to DAPI+ area using constant thresholding parameters across samples, using Fiji (ImageJ) version 1.54K. PSD95 results were obtained as the ratio of PSD95+ area to DAPI+ area. This approach provided a robust and reproducible measure of proliferative activity while minimizing segmentation bias.

Nuclear CTIP2 and SATB2 translocation levels were evaluated using Harmony Software (Revvity). Briefly, nuclear segmentation was performed to identify the nuclei population based on DAPI-guided masks, and an adjacent outer region was selected to delimit the cytoplasmic compartment. Protein fluorescence intensity was quantified in pixels for both nuclear and cytoplasmic regions. Nuclear translocation levels were then calculated by dividing the nuclear fluorescence by the cytoplasmic fluorescence, and results were expressed as arbitrary units. Image acquisition was performed with a PerkinElmer Operetta CLS high-content imaging system, optimized for broad-field coverage to ensure consistency and reproducibility across multiple fields and biological replicates. This approach prioritized sampling depth and quantitative robustness over subnuclear resolution, allowing objective assessment of CP-specific expression patterns across large sample areas.

### TUNEL assay

Cell apoptosis was evaluated on day-40 cerebral organoid cryosections using the *In Situ* Cell Death Detection Kit, TMR red (Roche, cat. no. 12156792910), following the manufacturer’s instructions with minor adaptations.

Organoids cryosections were fixed in acetone, permeabilized with 0.5% Triton for 15 min then incubated with the TUNEL reaction mixture (Enzyme Solution + Label Solution) for 1 h at 37 °C in a humidified chamber. After washing in PBS, nuclei were counterstained with DAPI and mounted with antifade medium.

### Immunoblotting

Brain organoid protein contents were determined by Western blotting. Organoids were lysed in ice-cold RIPA lysis buffer (89900, Thermo Fisher Scientific, MA, US) adding protease and phosphatase inhibitors. Samples were then sonicated and centrifuged (14,000 × g) for 20 min at 4˚C. The protein concentration of each sample was determined in triplicate with a Pierce™ BCA Protein Assay Kits (23225, Thermo Fisher Scientific, MA, US). Equal amounts of protein (15 ug) were separated by pre-cast 4-15% gels (Bio-Rad Laboratories, CA, US) and transferred to a PVDF membrane (Millipore A/S, Copenhagen, Denmark). Afterward, No-Stain™ Protein Labeling Reagent (A44449, Thermo Fisher Scientific, MA, US) was used to check for total protein loading and transfer efficiency. After blocking in TBS-Tween 3% BSA, membranes were incubated overnight with the primary antibody at 4 °C, and then with the appropriate HRP-conjugated secondary antibody for 1 h. Bands were visualized with ECL (Millipore) and recorded with a digital camera (iBright, Thermo Fisher Scientific, MA, US). Bands were quantified using Image Lab version 6.0 (Bio-Rad Laboratories) and determined as the total band intensity adjusted for background intensity and normalized for beta-actin.

Primary antibodies used were anti-total OXPHOS (ab110411, Abcam, Cambridge, UK), anti-beta actin (MA1-140, Thermo Fisher Scientific, MA, US), anti-synaptophysin (SAB4502906, Sigma-Aldrich, St. Louis, Missouri, US), anti-TBR1 (WH0010716M1, Sigma-Aldrich, St. Louis, Missouri, US), anti-Ctip2 (ab233713, Abcam, Cambridge, UK), anti-SATB2 (ab34735, Abcam, Cambridge, UK), anti-transthyretin (MA5-32634, Thermo Fisher Scientific, MA, US), anti-ER alpha (ab76228, Abcam, Cambridge, UK), anti-ER beta (ab187291, Abcam, Cambridge, UK), anti GPER (ab39742, Abcam, Cambridge, UK), anti-pAkt (4058S, Cell Signaling, Danvers, MA, US), anti-Akt (4691S, Cell Signaling, Danvers, MA, US), anti-pERK1-2 (9101, Cell Signaling, Danvers, MA, US), anti-ERK1-2 (9102, Cell Signaling, Danvers, MA, US), anti-pmTOR (ab109268, Abcam, Cambridge, UK), and anti-mTOR (PA534663, Thermo Fisher Scientific, MA, US).

The secondary antibodies used were HRP-conjugated goat anti-rabbit IgG (31460, Thermo Fisher Scientific, MA, US), and goat anti-mouse IgG (31430, Thermo Fisher Scientific, MA, US).

### Statistical analysis

The one-way analysis of variance (ANOVA) was used to check for significant differences when the only independent variable was represented by the treatments (organoids circularity, VLZ area, all immunofluorescence, and western blot data). When ANOVA showed significant results, Tukey’s *post-hoc* analysis was used to check for differences between the control and treatments. For organoids area and proliferation index, a mixed-effect analysis was used. Time and treatment were considered as independent variables and area or proliferation index as outcome measures. In this case, Fisher’s Least Significant Difference (LSD) test was used as a *post-hoc* analysis. Results were considered significant when p<0.05. All statistical analyses were performed using GraphPad PRISM version 10.2.1 (GraphPad Software, LLC).

## Results

### Chronic exposure to BPS affects size but not shape of developing brain organoids

**Figure 1:**
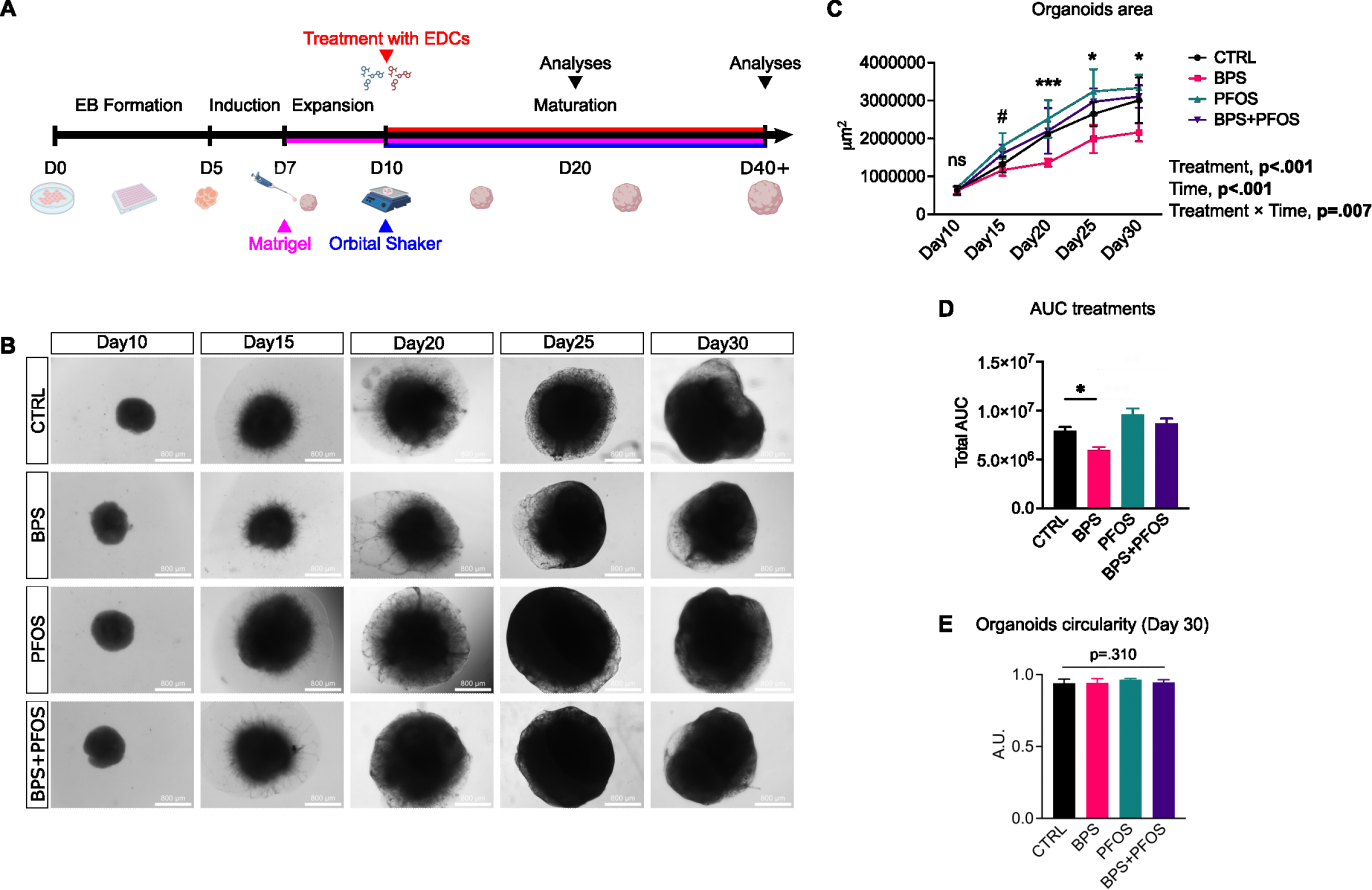
Effect of BPS and/or PFOS on the growth of brain organoids. **(A)** Schematic of the cerebral organoids differentiation timeline. **(B)** Representative brightfield microscopy images of the organoids from day 10 to day 30. Scale bar, 800 µm, 4x magnification. **(C)** Measures of the organoid area during the cell culture expressed in μm^2^, and mixed-effect analysis results. * p<.05, *** p<.001CTRL vs. BPS, and # p<.05 CTRL vs. PFOS; ns, non-significant. **(D)** Quantification of the AUC of different treatments over time. **(E)** Measures of organoid circularity at day 30. Organoid area and circularity results are based on n = 4 independent (2 for each cell line) organoid batches (biological replicates), each derived from independent differentiation runs, and that for each batch, at least n = 3 organoids were analyzed. Data are reported as mean and standard deviation.

To analyze the effects of environmental xenobiotics on the early and critical phases of human neurodevelopment, brain organoids were chronically exposed to BPS (50 nM), PFOS (100 nM), or a combination of both. These doses fall within the range of concentrations found in various human fetal tissues and organs, and are therefore representative of the actual exposure of the developing fetus (43–45). The treatment phase started on day 10 of culture, when neuronal induction was completed, and organoids began maturation, continuing up to day 40. It has already been reported, indeed, that 40 to 60-day-old organoids recapitulate the mid-fetal brain (10–16 post-conception weeks) (46, 47). A scheme of the cell culture protocol is reported in **Figure 1A**.

The effects of the treatments on the external morphology (size and shape) of the brain organoids were analyzed. The organoid area was monitored during the first phases of the maturation process using brightfield images (**Figure 1B****).** Mixed-effect analysis showed that both time and treatments had significant effects on organoid growth (**Figure 1C**): indeed, the organoids kept on growing during the culture in all the experimental conditions, but the BPS-treated remained constantly smaller than the other samples, as confirmed by the area under the curve (AUC) analysis (**Figure 1D**).

Then, the organoid circularity was measured; this parameter is used in organoid morphological studies because its changes often accompany alterations in the maturation process (48). Treatments did not affect the external shape of the organoids, as no changes were detected in the circularity values. (**Figure 1E****).**

### BPS and PFOS influence VLZ and neural stem cell proliferation inside the brain organoid

**Figure 2:**
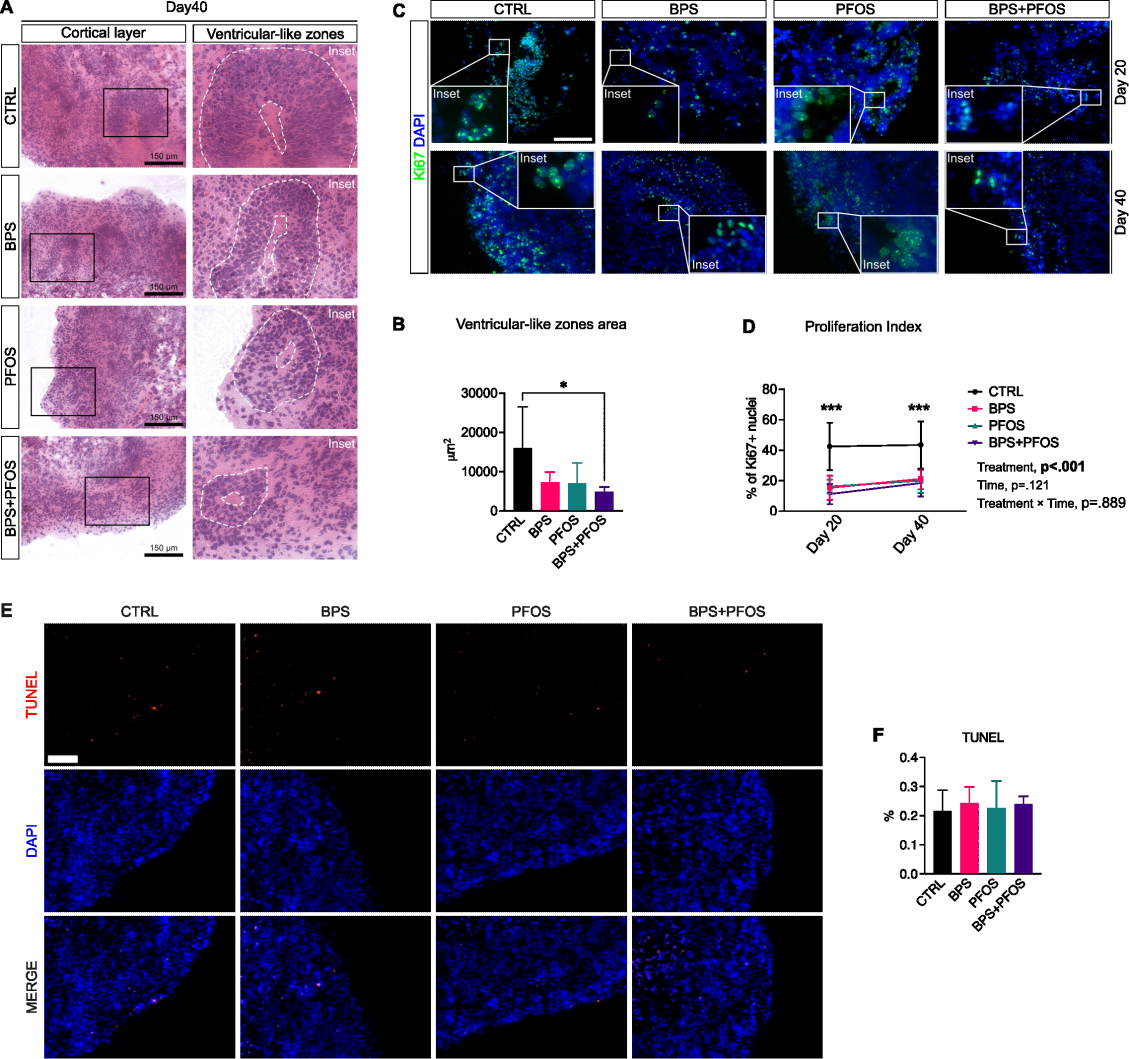
**(A)** Hematoxylin/eosin staining of cerebral organoids at day 40. Scale bar, 150 µm, original magnification 20 ×; the insets highlight representative VLZ structures. **(B)** Area of the VLZ expressed in μm^2^ at day 40. * p<.05. **(C)** Immunostaining of cerebral organoids for Ki67 (green fluorescence) at day 20 (upper row) and 40 (lower row). Nuclei are counterstained with DAPI (blue fluorescence). Scale bar, 150 µm, original magnification 20 ×. **(D)** Percentage of Ki67^+^ nuclei at days 20 and 40; the main mixed-effects analysis results are also reported. **(E)** Immunostaining of cerebral organoids for TUNEL (red fluorescence). Nuclei are counterstained with DAPI (blue fluorescence). Scale bar, 100 µm, original magnification 20 ×**(F)** Percentage of TUNEL^+^ nuclei at day 40 in the different condition analyzed by one-way ANOVA. VLZ area, proliferation index and TUNEL^+^ nuclei results are based on n = 4 independent (2 for each cell line) organoid batches (biological replicates), each derived from independent differentiation runs, and that for each batch, at least n = 3 organoids were analyzed with at least n = 5 fields per organoid. *** p<.001 CTRL vs treatments. In all graphs, data are reported as mean and standard deviation.

The developing human brain is characterized by ventricle-like cavities (ventricular-like zone – VLZ), proliferative regions of neural stem cells that, over time, move outward, producing the multilayered cortical-like structure. We then analyzed whether antenatal exposure to BPS and/or PFOS can induce abnormalities in the VLZ of the developing brain. Based on morphological features and spatial marker distribution, these VLZ structures were identified as round, densely nucleated regions located beneath the cortical-like plate. **Supplementary Figure S1** shows that Ki-67+ proliferative progenitors are predominantly localized within these internal areas, whereas βIII-tubulin (Tuj1) expression is enriched in the surrounding cortical regions, suggesting a spatial segregation between proliferating and post-mitotic cells consistent with early cortical development. At the end of the maturation period (day 40), brain organoids underwent hematoxylin and eosin staining, and the VLZ were analyzed. Data evidenced that the average VLZ area was smaller in BPS+PFOS samples compared to the control (**Figures 2A, B****).**

Then, to quantify the proliferation index of the neuronal progenitors, we examined the expression of Ki-67, a typical cell proliferation marker, by immunofluorescence. Analyses were performed on 20-day and 40-day organoids to monitor the effect of the treatments during the maturation period. As expected, proliferative zones were predominantly detected at the cortical level and within the VLZ (**Figure 2C****).**

The quantification of Ki-67+ nuclei revealed that, under all experimental conditions, the proliferation rate remained constant throughout the maturation phase, with no significant change observed between day 20 and day 40. However, control organoids showed a significantly higher percentage of Ki-67+ nuclei compared to those exposed to BPS, PFOS, and BPS+PFOS, both on day 20 and 40 (**Figure 2D****).**

Since reductions in Ki-67+ cells could reflect either impaired proliferation or increased cell death, we performed a TUNEL assay at day 40 to assess whether the observed effects might result from unspecific cytotoxicity (**Figure 2E**). The analysis did not reveal any statistically significant increase in apoptotic cells across treatment groups compared to controls (**Figure 2F**), indicating that chronic low-dose exposure to BPS, PFOS, or their combination did not induce measurable apoptosis in this system.

Taken together, these findings suggest that the decrease in proliferating progenitors is unlikely due to overt cytotoxicity but reflects specific alterations in neurodevelopmental programs induced by chronic exposure to environmentally relevant concentrations of BPS and PFOS.

### BPS and/or PFOS interfere with the development of cerebral cortical layers and choroid plexus formation

**Figure 3:**
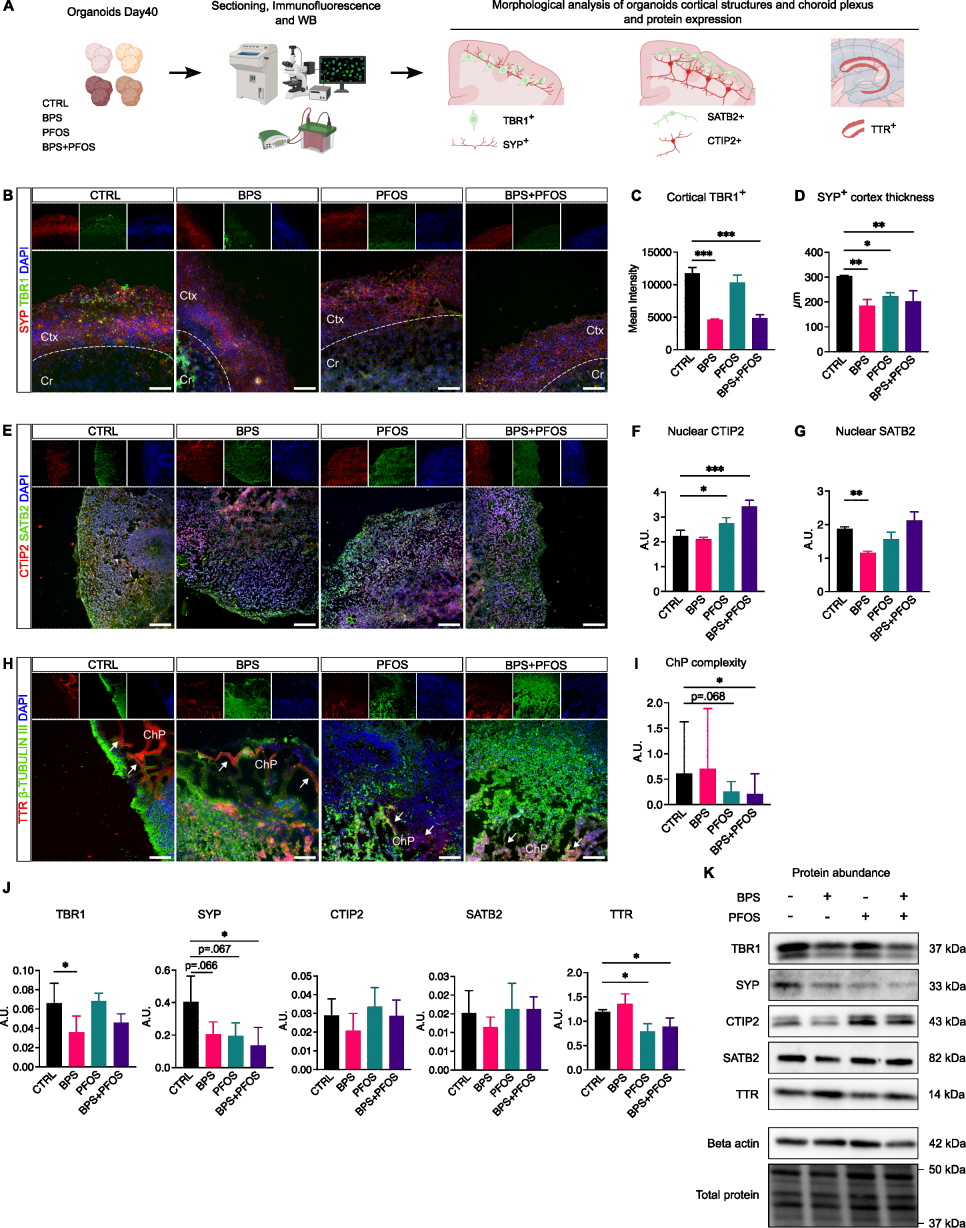
Effect of BPS and/or PFOS on brain organoid architectures. **(A)** Experimental procedures and workflow. **(B)** SYP (red fluorescence) and TBR1 (green fluorescence) localization in control and treated brain organoids; the cortical and central zones are divided by the dashed line. **(C)** Quantification of TBR1 expression at cortical level. **(D)** Quantification of SYN positive cortical thickness. **(E)** Representative images of CTIP and SATB2 localization. **(F, G)** Quantification of nuclear levels of CTIP2 and SATB2. **(H)** TTR (red fluorescence) and β-Tubulin III (green fluorescence) expression. White arrows indicate the TTR^+^ zone surrounding the cavities. **(I)** Measures of choroid plexus complexity. **(J, K)** Western blot images and band quantification of TBR1, SYP, SATB2, CTIP2, and TTR expression in the different experimental conditions. Microscopic images results are based on n = 4 independent (2 for each cell line) organoid batches (biological replicates), each derived from independent differentiation runs, and that for each batch, at least n = 3 organoids were analyzed with at least n = 5 fields per organoid. Western blot results are based on n = 4 independent (2 for each cell line) organoid batches (biological replicates), each derived from independent differentiation runs, and for each batch, n = 3 organoids were pooled. In the immunofluorescence, nuclei are counterstained with Dapi (blue fluorescence). Scale bar 100 µm, original magnification: 20 ×; Ctx, cortex; Cr, central region; ChP, choroid plexus. In all graphs, data are reported as mean and standard deviation. *p<.05, **p<.01, ***p<.001.

We then analyzed the effect of BPS and/or PFOS exposure on the internal architecture of brain organoids, focusing on the development of the cortical plate (CP) and the formation of the choroid plexus (ChP). To assess cortical development, we first examined T-box brain 1 (TBR1) and synaptophysin (SYP) expression in 40-day-old brain organoids (**Figure 3B**). TBR1 is a transcription factor of the T-box family, expressed soon after cortical progenitors begin differentiating, and it is highly detected in early-born neurons of the preplate (49). Immunofluorescence analysis showed that TBR1 expression was reduced by BPS and BPS+PFOS treatment compared to the control, while no differences were observed when cells were exposed to PFOS alone (**Figure 3C**). Western blot analysis confirmed a decrease in TBR1 expression following BPS exposure compared to control (**Figures 3J, K****).**

We then focused on SYP, a synaptic vesicle membrane protein expressed throughout the brain and widely used as a marker of synaptogenesis (50). In 40-day organoids, SYP signal was detected primarily in the cortical region, where newly differentiated neurons begin to establish synaptic connections (**Figure 3B**). Consistent with its known expression pattern during early development, SYP immunoreactivity was not restricted to discrete synaptic puncta but showed a diffuse distribution along neuronal process. This pattern is typical of immature neurons and reflects ongoing synaptic assembly and vesicle transport, as reported in previous studies (51, 52). The thickness of the SYP-positive zone, corresponding to the CP, was significantly reduced in all treated groups, with the most pronounced decrease observed following BPS and BPS+PFOS exposure (**Figure 3D****).** Western blot quantification of SYP confirmed the downregulation of this protein, particularly in the BPS+PFOS group (**Figures 3J, K****).** To strengthen these findings, we performed an additional co-staining for PSD95, a canonical postsynaptic marker, together with Tuj1, which labels the neuronal cytoplasm. The PSD95 signal was predominantly localized in the cortical regions and showed a substantial reduction in treated organoids, consistent with the observed SYP pattern (**Supplementary Figure S2**). These findings indicate that both presynaptic (SYP) and postsynaptic (PSD95) compartments are impaired by chronic exposure to BPS and PFOS, supporting the hypothesis of altered synaptogenesis and delayed cortical maturation.

**Figure 4:**
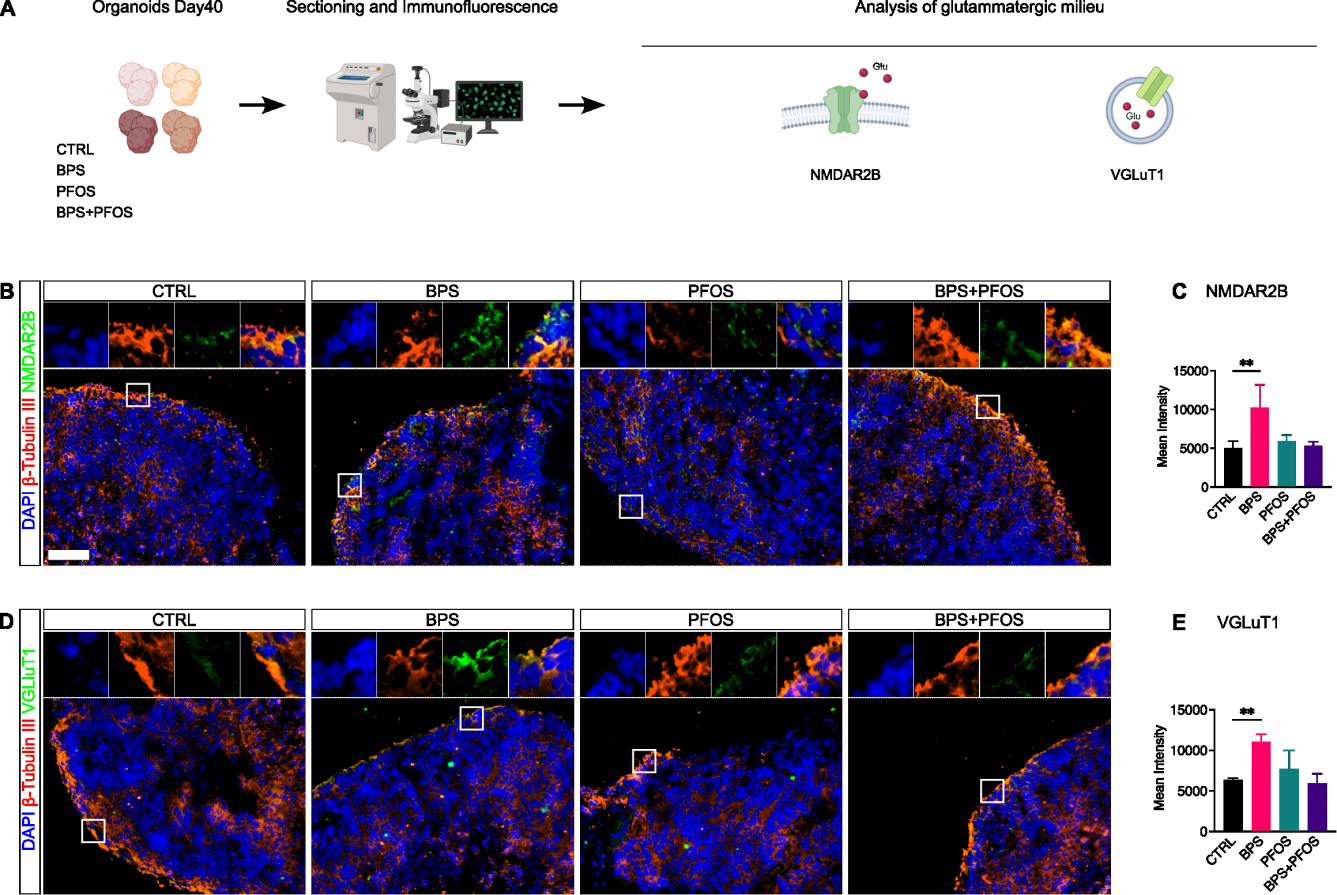
Effect of BPS and/or PFOS on NMDAR2B and VGluT1 expression in 40-days organoids. **(A)** Experimental procedures and workflow. **(B-E)** Expression and quantification of NMDAR2B and VGluT1 by immunofluorescence. In all graphs, data are reported as mean and standard deviation. *p<.05, **p<.01, ***p<.001. Microscopic images results are based on n = 4 independent (2 for each cell line) organoid batches (biological replicates), each derived from independent differentiation runs, and that for each batch, at least n = 3 organoids were analyzed with at least n = 5 fields per organoid. Nuclei are counterstained with DAPI (blue fluorescence) while neural cytoskeleton with β-Tubulin III (red fluorescence). Scale bar, 100 µm, original magnification 20 ×. White square indicates the region magnified in the inset.

We then analyzed the effects of BPS and/or PFOS on neuronal specification within the CP. In the developing mammalian neocortex, the thickness of CP increases, allowing the six specialized cortical layers to develop, with a specific layer-dependent expression of neuronal subtype markers (53). Specifically, we focused on COUP TF1-interacting protein 2 (CTIP2) and SATB homeobox 2 (SATB2): initially co-expressed by early born cortical neurons, the expression of these molecules becomes more selective during CP maturation. SATB2 predominantly marks intracortical projection neurons in the upper layers (II-IV), while CTIP2 identifies subcortical projection neurons in deeper layers (V-VI). Immunofluorescence analysis showed that in day-40 brain organoids, SATB2 was mainly localized in the outermost layers of the CP, whereas CTIP2 labeling was more diffuse, mainly staining the inner zones. This maturation pattern was altered by the treatments, as in all treated samples, the separation between SATB2+ and CTIP2+ populations was less clear, showing a mixed positivity without the layered localization seen in controls (**Figure 4E**). Being transcription factors, CTIP2 and SATB2 translocate into the nucleus to interact with DNA. Thus, we quantified the nuclear expression of these markers: results showed that organoids treated with PFOS and the BPS+PFOS combination had significantly increased nuclear CTIP2 compared to controls (**Figure 4F**). Conversely, BPS treatment reduced nuclear SATB2 content relative to controls (**Figure 4G**). No differences in total CTIP2 and SATB2 expression were detected via Western blot (**Figures 4J, K**). These findings suggest that BPS and PFOS may impair the normal processes of CP formation and neuronal specification, affecting TRB1 expression and disrupting the nuclear translocation of SATB2 and CTIP2.

We finally analyzed the effects of BPS and/or PFOS exposure on the ChP formation. It has already been reported that day-40 brain organoids display internal areas of columnar or cuboidal cells enriched in transthyretin (TTR) that delimit small cavities in which the cerebrospinal fluid is secreted (54). Thus, to analyze ChP development, immunofluorescent analysis was performed by labeling brain organoid slices for β-Tubulin III, an early marker of neural differentiation, and for TTR, which identifies the ChP. In our models, the EDCs dramatically interfere with the ChP development, as evidenced by a weaker TTR fluorescence and cavities of minor dimensions in the treated samples (**Figure 3H**). To confirm this observation, we measured the “complexity” of ChP architecture; ChP complexity was calculated as luminal area divided by total ChP area (i.e., the area delineated by TTR^+^ staining). This parameter reflects the degree of folding and branching of the ChP epithelium, which is a morphological hallmark of its maturation and secretory specialization. Results evidenced BPS+PFOS treatment significantly affected the development of the ChP, changing its architecture (**Figure 3I**). Finally, Western blot analysis confirmed the downregulation of TTR protein expression after PFOS and BPS+PFOS treatments compared to control (**Figures 3J, K****).**

### BPS exposure affects glutamatergic neurogenesis

The formation of the human cerebral cortex *in vivo* involves the assembly of canonical circuits, with glutamate being the principal excitatory neurotransmitter (53). To investigate the effect of EDCs on glutamatergic neurogenesis, we examined NMDAR2B and VGluT1 expression through immunofluorescence analysis. NMDAR2B is a subunit of NMDA-type glutamate receptors that localizes at both postsynaptic and extra-synaptic sites, including neurites and soma, and plays a central role in neuronal excitability, calcium signaling, and synaptic plasticity (55, 56). These markers were co-stained with βIII-tubulin (Tuj1) to better visualize their localization and distribution.

**Figure 5:**
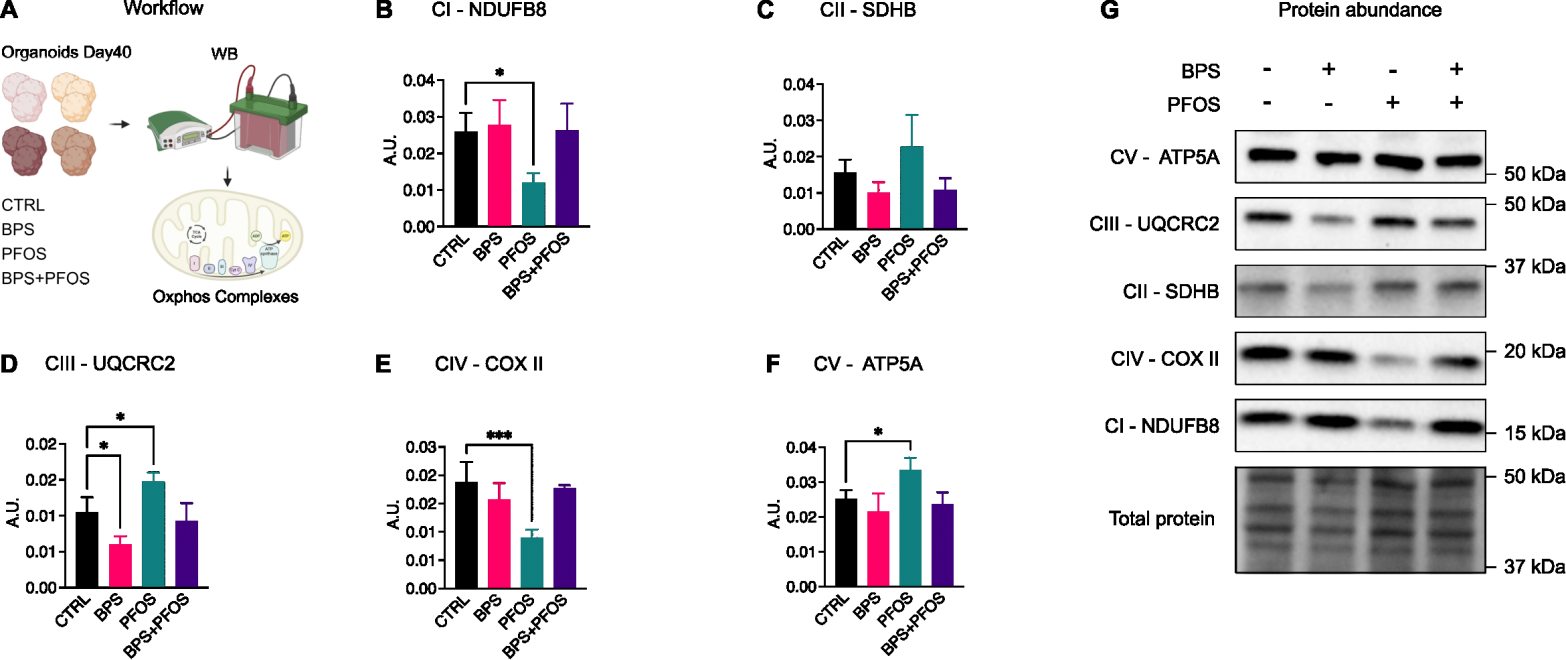
Effect of BPS and/or PFOS on the ETC complexes proteins. **(A)** Schematics of the experimental workflow: on day 40, brain organoids were processed for Western blot analysis and the expression of the five ETC complexes was evaluated, **(B-G)** Western blot images and band quantification of Complex I (NDUFB8), Complex II (SDHB), Complex III (UQCRC2), Complex IV (COXII), Complex V (ATP5A). Western blot results are based on n = 4 independent (2 for each cell line) organoid batches (biological replicates), each derived from independent differentiation runs, and for each batch, n = 3 organoids were pooled. In all graphs, data are reported as mean and standard deviation. *p<.05, ***p<.001.

Both NMDAR2B and VGluT1 immunoreactivity were detected primarily within the CP (**Figures 5B, D**), where newly generated glutamatergic neurons begin to form synaptic connections. This cortical localization is biologically consistent with the developmental role of these markers in excitatory circuit assembly.

Both antigens exhibited a partially clustered positivity, consistent with their expression pattern during early synaptic development (57, 58). Quantitative analysis of fluorescence intensity revealed that BPS exposure, unlike PFOS, significantly increased NMDAR2B and VGluT1 expression, suggesting an EDC-driven modulation of glutamatergic maturation (**Figures 5C, E****).**

### BPS and PFOS differentially regulate the expression of the mitochondrial oxidative phosphorylation complexes

To determine whether the morphological and molecular modifications observed upon xenobiotic treatment were also accompanied by metabolic alterations in developing neurons, an analysis of mitochondrial oxidative phosphorylation (OXPHOS) complexes was performed. OXPHOS represents the primary energy source necessary for cellular functions, and mitochondrial activity plays a key role in neuronal development, axiogenesis, and synaptogenesis (59).

Day-40 brain organoids were lysed, and the expression of electron transport chain (ETC) complexes was assessed by Western blot analysis (**Figure 5A**). Results indicated that exposure to BPS and/or PFOS modulated the expression of several OXPHOS components, although with distinct compound- and complex-specific profiles (**Figure 5G**). Specifically, PFOS exposure reduced Complex I (NADH dehydrogenase ubiquinone 1 beta subcomplex subunit 8 – NDUFB8, **Figure 5B**) and Complex IV (cytochrome c oxidase subunit 2 – COX II, **Figure 5E**) protein levels, while increasing Complex III (ubiquinol-cytochrome c reductase core protein 2 - UQCRC2, **Figure 5D**) and Complex V (ATP synthase lipid-binding protein - ATP5A, **Figure 5F**) expression. In contrast, BPS treatment led to a reduction in Complex III compared to control (**Figure 5D**). The expression of Complex II (succinate dehydrogenase complex II, subunit B - SDHB) was not significantly regulated by treatments (**Figure 5C**).

### Chronic exposure to BPS, but not PFOS, disrupts genomic and non-genomic estrogen signaling pathways

To explore potential mechanisms underlying the morphological and metabolic alterations previously observed, we investigated estrogen signaling pathways, because a growing body of evidence suggests that EDCs including bisphenols and perfluoroalkyl substances, may exert their effects by interfering with estrogen-mediated signal transduction. Both genomic and non-genomic estrogen signaling routes have been implicated in neurodevelopment and may be considered susceptible targets for xenoestrogenic compounds. The genomic pathway involves classical nuclear estrogen receptors (ERα and ERβ), which, upon ligand binding, act as transcription factors to regulate gene expression programs essential for neurodevelopment. In contrast, the non-genomic pathway is initiated by membrane-bound receptors, notably G protein-coupled estrogen receptor 1 (GPER), and leads to rapid intracellular responses through activation of signaling cascades such as ERK1/2 and Akt–mTOR.

**Figure 6:**
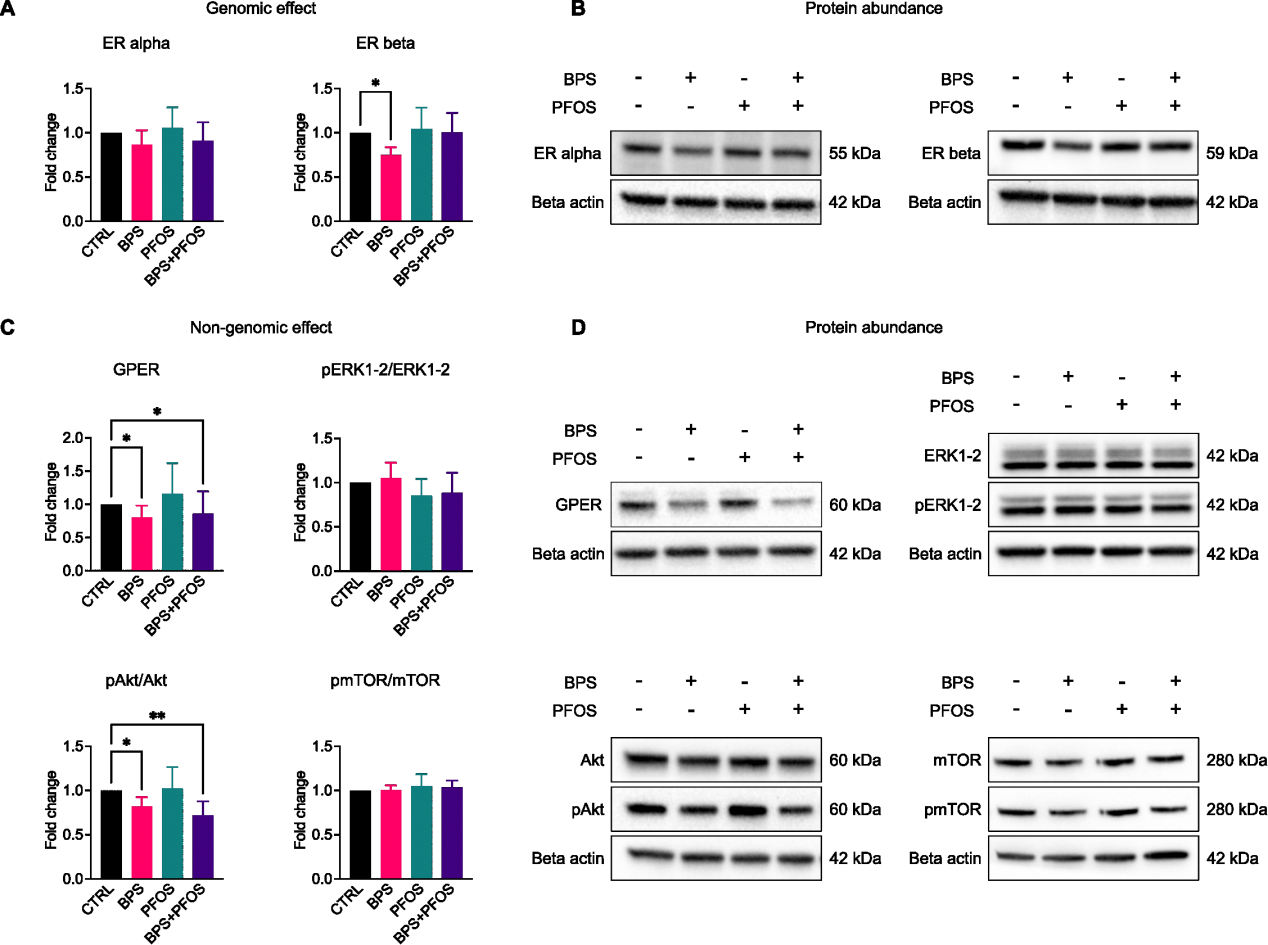
Effects of BPS and/or PFOS on genomic and non-genomic cascades. Western blot images and band quantification of ER alpha, ER beta **(A, B),** GPER, ERK1-2, Akt, and mTOR **(C, D)**. Western blot results are based on n = 8 independent (4 for each cell line) organoid batches (biological replicates), each derived from independent differentiation runs, and for each batch, n = 3 organoids were pooled. Results were obtained from 8 independent experiments (4 for each cell line), 3 organoids were pooled for each condition. * p<.05, ** p<.01.

Results showed that only BPS treatment interfered with the genomic signaling pathway, resulting in a significant decrease in ERβ expression (**Figures 6A, B****).** Conversely, analysis of the non-genomic pathway revealed a downregulation of GPER expression following exposure to BPS and the BPS+PFOS combination (**Figures 6C, D**). As the key downstream effectors of this pathway ERK1/2 and Akt–mTOR signaling cascades, rely on phosphorylation for activation, we analyzed their phosphorylation levels. Results evidenced that pERK1/2 remained unaffected by any treatment, while a reduction in pAkt levels was observed after exposure to BPS and BPS+PFOS. In contrast, mTOR phosphorylation showed no significant change (**Figures 6C, D**).

## Discussion

Concerns are mounting about the immediate and long-term consequences of human exposure to EDCs such as BPS and PFOS. These compounds interfere with hormonal signaling and may compromise developmental processes and long-term health. Pregnancy is a particularly vulnerable period, as both chemicals cross the placental barrier and reach fetal tissues, potentially causing lasting alterations.

Several prospective and epidemiological studies have linked prenatal EDC exposure to disruptions in neurodevelopmental trajectories, with potential consequences for cognition, emotional regulation, and social behavior (60, 61). Evidence from animal models supports these associations (62), though interspecies differences in neuroendocrine development limit their predictive value (29). By evaluating BPS and PFOS both individually and in combination at concentrations within the range detected in pregnant women’s serum (63, 64), this study leverages a human brain organoid model to replicate realistic exposure scenarios. This approach not only differentiates compound-specific from interaction-driven effects but also provides a biologically relevant framework to elucidate whether chronic co-exposure can intensify neurodevelopmental risk.

This study investigated the impact of chronic low-dose exposure to BPS and PFOS on selected aspects of early human brain development, using a cerebral organoid model. By examining structural, cellular, and molecular parameters, we identified several exposure-related changes suggestive of altered neurodevelopmental trajectories.

Our data show that both BPS and PFOS affect brain organoid growth during maturation, without altering overall morphology but causing a slight reduction in size, particularly in BPS-treated samples. This reduction appears to reflect decreased proliferative activity within the VLZ, the neuroepithelial region containing precursors for cortical excitatory neurons. Organoids exposed to BPS and/or PFOS exhibited lower expression of the proliferative marker Ki67, suggesting disruption of the neurogenic timeline. Importantly, TUNEL assay did not reveal any increase in apoptosis across experimental conditions, indicating that the reduction in Ki67 expression is not attributable to cytotoxicity but rather to altered proliferation. These findings confirm and expand our previous data (5–7) highlighting that even low-dose exposure to bisphenols or perfluoroalkyls can alter the proliferative rate and biological characteristics of stem cells present in both the developing fetus and fetal annexes. Since these progenitors must tightly regulate the balance between self-renewal and differentiation, early interference may induce long-lasting alterations in brain development.

Starting from the VLZ, proliferative neural stem cells migrate outward to generate the multilayered CP, composed of deep- and superficial-layer neurons (65). In BPS-exposed organoids, we observed a reduction in TBR1 expression, a transcription factor critical for early-born neuron identity and cortical layer organization (66). Proper regulation of TBR1 is essential for cortical development, and its downregulation suggests altered CP maturation. Whether due to fewer neurons populating the cortical plate or to lower TBR1 expression per cell, both interpretations converge on a biologically relevant impairment in CP formation. In parallel, we found reduced expression and thickness of SYP-positive areas across all treated samples, indicating impaired synaptogenesis. Since SYP marks synaptic density (67), its reduction reinforces the idea that BPS and PFOS interfere with neuronal connectivity. This observation was further supported by the decreased expression of PSD95, a canonical postsynaptic marker, whose distribution pattern and intensity closely mirrored that of Synaptophysin. Together, these two markers point to a converging effect on both presynaptic and postsynaptic compartments, suggesting a delay or impairment in the formation of functional synapses. These findings align with prior studies showing that BPA, structurally similar to BPS, disrupts synaptogenesis and induces behavioral alterations in animal models (68). Despite BPS being marketed as a safer alternative to BPA, it is often detected at higher concentrations in both maternal and fetal samples (69, 70) raising concerns about its safety profile. Recent work in rodents further supports our findings, demonstrating that BPS impairs hippocampal neurogenesis and newborn neuron maturation (71).

The effects of BPS and PFOS on cortical plate development extend beyond proliferation and early neurogenesis, involving alterations in neuronal subtype specification. SATB2 and CTIP2 are transcription factors essential for the development of intracortical and subcortical projection neurons, respectively (72). While Western blot analysis did not reveal changes in their expression levels, morphological analysis showed a disrupted laminar organization in treated organoids. In controls, SATB2 and CTIP2 displayed distinct layer-specific localization, with SATB2 labeling upper CP layers and CTIP2 marking deeper strata. In contrast, EDC-treated samples exhibited a more diffuse distribution, indicating impaired neuron positioning and specification.

To further explore transcription factor activity, we employed high-content imaging and broad-field acquisitions, which enabled quantitative evaluation of nuclear versus cytoplasmic signal across thousands of cells. Although this method does not capture fine subnuclear details, it provides an effective readout of transcription factor localization dynamics and supports robust population-level comparisons. PFOS, alone or in combination with BPS, increased nuclear translocation of CTIP2, suggesting enhanced subcortical neuron fate at the expense of callosal projections. Conversely, BPS exposure was associated with reduced nuclear SATB2 levels, hinting at compromised development of commissural neurons. These shifts in cortical identity may contribute to aberrant brain connectivity, a hallmark of several neurodevelopmental disorders (73).

The ChP, responsible for producing cerebrospinal fluid (CSF), plays a crucial role in maintaining brain homeostasis during development (74). The main protein synthesized and secreted by the ChP is transthyretin (TTR), which is essential for the transport of thyroid hormones from the blood into the CSF (75). Reduced TTR expression and the morphological alterations of the TTR+ regions, as observed in PFOS-exposed organoids, may contribute to impaired thyroid hormone delivery to the developing CNS. Since thyroid hormones are essential for neural progenitor proliferation, cortical layering, and synapse formation, this decrease in TTR may help explain the concurrent deficits observed in neurogenesis, cortical plate maturation, and synaptic development within our model. Disruption of ChP development may thus have widespread consequences, including impaired CSF production, altered barrier integrity, and disrupted signaling between the brain and peripheral organs. These findings are consistent with prior evidence showing that EDCs can affect the blood–brain and blood–CSF barriers and impair CSF production, potentially contributing to neurodevelopmental disorders (76). Given the essential role of thyroid hormones in normal brain development, even subtle alterations in TTR expression may influence thyroid hormone availability to the CNS during fetal growth, with long-term consequences. While this association is biologically plausible, further studies are needed to determine whether and how such changes functionally impact thyroid hormone–dependent neurodevelopmental processes.

Glutamate is the main excitatory neurotransmitter in the central nervous system, and glutamatergic signaling is fundamental for synaptic plasticity, learning, and memory (77, 78). In our model, organoids exposed to BPS showed increased expression of NMDAR2B and VGluT1, suggesting an association with enhanced glutamatergic marker expression during early differentiation. While this may suggest an accelerated excitatory differentiation program, whether these molecular alterations translate into functional changes in glutamatergic connectivity remains to be investigated. Our findings align with previous studies reporting that EDCs, such as BPA, a structural analog of BPS, can enhance glutamatergic activity and disrupt synaptic dynamics *in vivo* (79). Likewise, BPA exposure was associated with increased VGluT1 expression in rodent models, possibly contributing to aberrant synaptic function (80). These results support the idea that BPS, though introduced as a safer alternative to BPA, may exert comparable effects on excitatory neuronal pathways. Interestingly, while BPS alone increased expression of glutamatergic markers, co-exposure with PFOS attenuated this upregulation. Although the mechanistic basis of this modulation remains to be clarified, it highlights the importance of considering combined chemical effects in neurodevelopmental risk assessment. Indeed, mixtures of EDCs may lead to additive, synergistic, or antagonistic outcomes (5–7, 24, 81), complicating the prediction of their actual toxicological impact in real-life scenarios.

Mitochondria are central regulators of energy metabolism in the developing brain, supporting neurogenesis, synaptic formation, and plasticity. Alterations in mitochondrial protein expression have been implicated in various neurodevelopmental and neurodegenerative conditions. In our study, BPS and PFOS exposure resulted in distinct alterations in the expression of proteins from the mitochondrial electron transport chain (ETC), suggesting that these compounds may modulate mitochondrial regulatory pathways during neurodevelopment. Specifically, PFOS-treated organoids showed downregulation of Complex I and Complex IV. While we did not assess functional respiration, this expression pattern is consistent with previous studies reporting PFOS-related effects on mitochondrial biology and redox homeostasis (82, 83). Concurrently, PFOS induced an upregulation of Complex III and Complex V components, which could reflect a compensatory shift in mitochondrial protein regulation. BPS-treated organoids exhibited a different profile, with decreased expression of Complex III subunits. Similar findings have been described in studies showing BPS-associated alterations in mitochondrial protein expression and oxidative stress marker (84). Notably, in the combined BPS+PFOS condition, the ETC expression pattern more closely resembled that of BPS alone, suggesting potential dominance of BPS-mediated effects under co-exposure conditions. Overall, these findings highlight that BPS and PFOS can differentially influence the expression of mitochondrial ETC proteins. While these observations point to a potential role for mitochondrial signaling in EDC-induced neurodevelopmental effects, further functional studies are required to determine whether these molecular changes impact energy metabolism or oxidative balance in developing neurons.

Estrogens play a crucial role in brain development, regulating key processes such as neuronal proliferation, migration, and synapse formation. Given the hormone-sensitive nature of the developing brain, prenatal exposure to EDCs may interfere with critical neurodevelopmental pathways. Based on previous evidence indicating that bisphenols and perfluoroalkyls can act as xenoestrogens (85, 86), we investigated whether the morphological and metabolic alterations observed in our model might be associated with disruptions in estrogen signaling. In our study, BPS exposure was associated with decreased expression of ERβ, a nuclear receptor involved in neurodevelopmental gene regulation, and with altered levels of GPER, the membrane-bound estrogen receptor known to mediate non-genomic signaling. In parallel, a reduction in Akt phosphorylation was observed in BPS-treated organoids, in the absence of significant changes in mTOR or ERK1/2 phosphorylation. These observations are consistent with a possible modulation of the GPER–PI3K/Akt axis. Notably, this pattern aligns with our recent findings in a different cellular model, where BPS and PFOS were shown to alter both genomic and non-genomic estrogen signaling in stem cells (12).

While these data suggest a potential involvement of estrogen receptors in mediating the effects of BPS, it is important to note that the PI3K/Akt and ERK1/2 pathways can also be regulated by ER-independent mechanisms, which may themselves be modulated by xenobiotics such as BPS or PFOS. Furthermore, the tissue-specific nature of GPER downstream signaling must be considered: in developing neural contexts, GPER may preferentially engage the Akt cascade while bypassing other canonical targets such as ERK1/2 or mTOR (87). These findings align with previous reports of EDC-mediated impairment of estrogen receptor activity in neuronal models (88), and reinforce the view of BPS as xenoestrogen with specific effects on non-genomic pathways, which might be particularly relevant in the context of early neurodevelopment, where even subtle hormonal imbalances may result in long-term structural and functional deficits (89). It is worth noting that activation of estrogen receptors can enhance mitochondrial biogenesis and regulate expression of oxidative phosphorylation complexes in neuronal and non-neuronal tissues (90, 91).

In our model, the observed reduction of OXPHOS protein expression after BPS exposure may therefore reflect, at least in part, disruption of estrogen receptor–mediated mitochondrial regulation. Overall, our results support a hypothesis-generating association between BPS exposure and altered estrogen receptor–linked signaling in human brain organoids. However, the lack of direct functional manipulation (e.g., use of receptor agonists/antagonists) precludes definitive mechanistic conclusions. In addition, an involvement of the Akt signaling axis independently of estrogen receptor activation cannot be excluded. Several environmental stimuli, including xenobiotics, are known to modulate Akt and ERK1/2 through alternative, estrogen-independent mechanisms, which may contribute to the observed changes in our model.

Although PFOS did not alter classical estrogen signaling markers in our model, its inclusion in the estrogen pathway assessment was necessary to verify potential crosstalk with estrogenic mechanisms, given previous reports of receptor-mediated activities for some PFAS. In our study, PFOS exposure did not produce direct changes in ER-related markers, suggesting a limited contribution of classical estrogen signaling to PFOS neurotoxicity in this system. Nonetheless, PFOS exposure profoundly affected brain organoid development, disrupting choroid plexus (ChP) formation and reducing transthyretin (TTR1) expression, alterations that may indirectly impact thyroid hormone availability during critical stages of neurodevelopment. This hypothesis aligns with the literature evidence indicating that PFOS can interfere with the thyroid hormone axis at multiple levels (92–94), potentially contributing to deficits in neurogenesis, synaptogenesis, and cognitive functions. The concordance between our findings and existing reports reinforces the plausibility of thyroid-related mechanisms in mediating PFOS effects. These observations also highlight that simultaneous exposure to multiple EDCs, each potentially targeting distinct hormonal axes such as estrogens and thyroid hormones, may result in complex neurodevelopmental disturbances. Such effects cannot be easily extrapolated from single-compound studies, reinforcing the relevance of mixture-based toxicological approaches.

## Study limitations

This study presents some limitations that should be acknowledged:

- the experiments were conducted exclusively using hiPSCs derived from male donors, which may limit the generalizability of our findings. Given the well-established influence of sex hormones on brain development, future studies should include female-derived hiPSCs to evaluate potential sex-specific responses to BPS and PFOS exposure.
- although the concentrations of BPS and PFOS used in our model are in the range of those detected in human biological fluids, the absence of a dose–response assessment limits our ability to define threshold levels for toxicity and to explore potential non-monotonic effects, which are common among EDCs.
- our analysis focused primarily on morphological and molecular endpoints, with particular attention to glutamatergic differentiation due to its known sensitivity to environmental toxicants and relevance in excitatory–inhibitory balance. However, functional consequences—such as altered electrophysiological activity, synaptic integration, or long-term neuronal network maturation—were not addressed in this study and require dedicated follow-up investigations.
- While our data support the involvement of estrogen signaling—based on the observed changes in ERβ and GPER expression and in Akt phosphorylation—we did not assess downstream transcriptional activity or specific gene targets, which would be necessary to fully characterize the genomic consequences of this pathway. In addition, the observed modulation of Akt and ERK signaling may also reflect activation of estrogen-independent mechanisms, which were not explored in the present study and should be considered in future investigations. Finally, the potential contribution of other endocrine axes, such as thyroid hormone signaling in PFOS-exposed samples, remains hypothetical and warrants direct functional validation to confirm its role in the observed neurodevelopmental phenotypes.
- Another limitation of our study is the absence of glial cell characterization. Although the genesis of glial cells in brain organoids typically emerges at later stages (>80–100 days) (95), we did not assess the presence or maturation of glial populations in our 40-day model. As a result, our findings are limited to neuronal development and do not capture potential EDC-related effects on astrocytes or other glial lineages.

## Conclusion

Our findings demonstrate that chronic exposure to environmental doses of BPS and PFOS interferes with key stages of human brain development in cerebral organoids, including progenitor proliferation, neuronal identity specification, synaptogenesis, and mitochondrial protein expression. BPS acted as a xenoestrogen, disrupting both genomic (ERβ) and non-genomic (GPER-Akt) estrogen signaling. PFOS, although not altering the estrogenic pathway, induced changes consistent with reduced availability of thyroid hormones to the developing CNS, as suggested by decreased TTR1 expression.

These data highlight that distinct EDCs can target different hormonal systems, with potential for converging or synergistic effects on neurodevelopment in the real-life mixed exposure. Low-dose exposure to EDCs can therefore produce biologically significant outcomes with possible long-term consequences, underscoring the need for precautionary approaches in their risk assessment. Our data provide mechanistic and dose-relevant evidence to inform regulatory strategies aimed at reducing early-life exposure to persistent and bio-accumulative pollutants.

## Data Availability

The raw data supporting the conclusions of this article will be made available by the authors, without undue reservation.
