# Supplementary figures and images for: Endocrine-disrupting effects of environmental BPS and PFOS on human brain organoid development
Source: Front Endocrinol (Lausanne). 2025 Dec 4;16:1692333. doi: 10.3389/fendo.2025.1692333 (PMC12711465; doi:10.3389/fendo.2025.1692333)

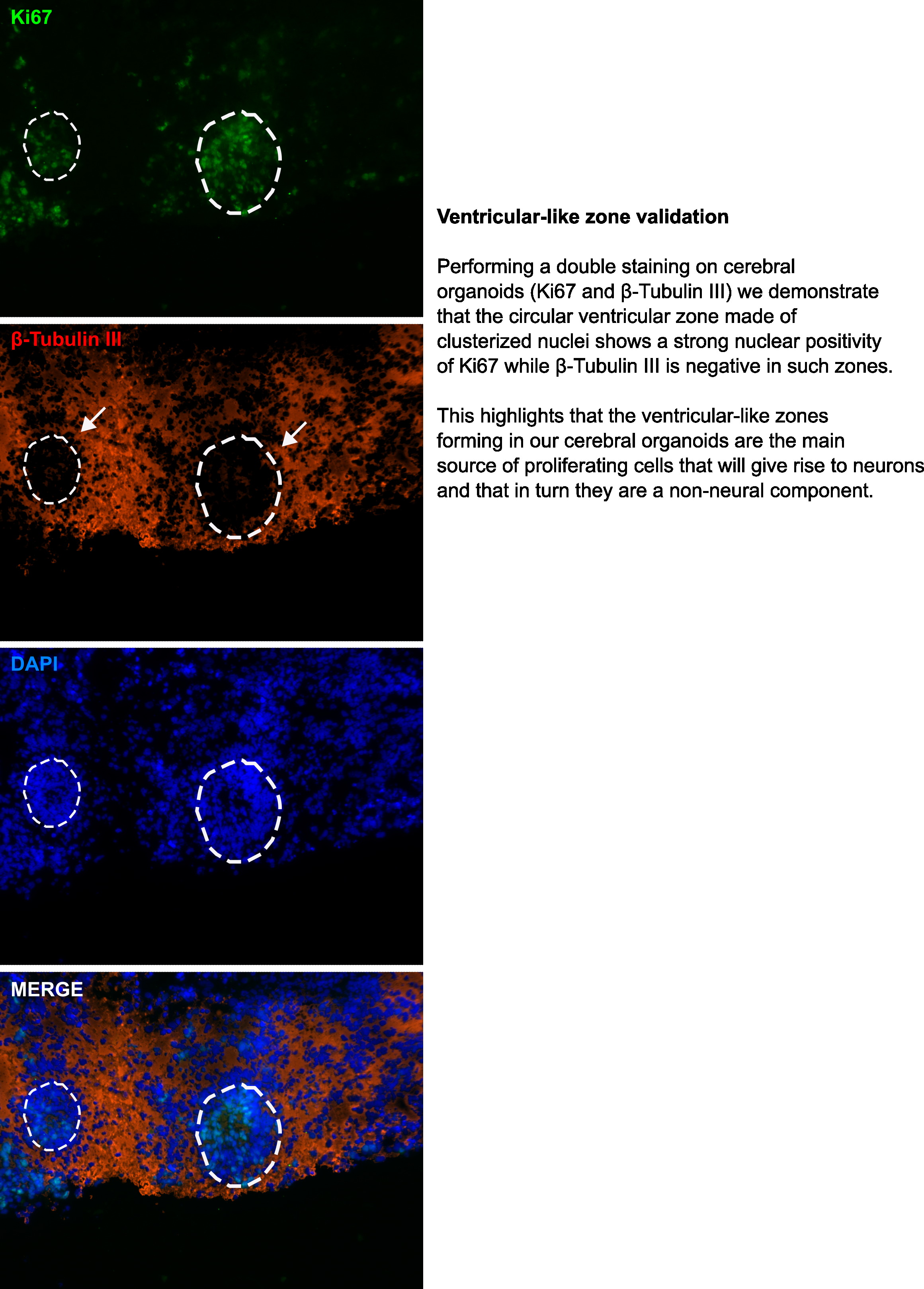

Supplement: Supplementary file 1 [file Image1.tiff]

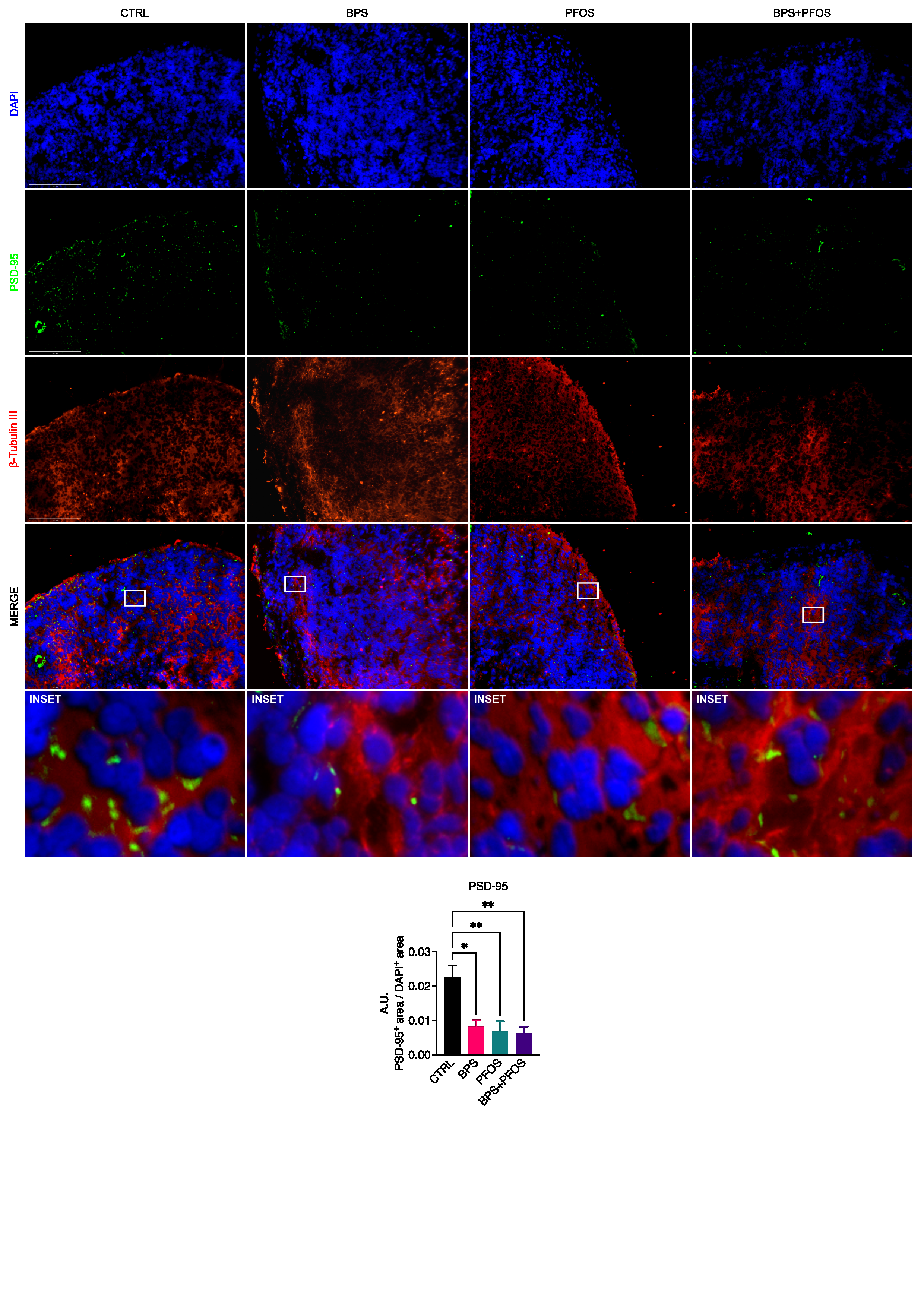

Supplement: Supplementary file 2 [file Image2.tiff]
